# FusionFinder: A Software Tool to Identify Expressed Gene Fusion Candidates from RNA-Seq Data

**DOI:** 10.1371/journal.pone.0039987

**Published:** 2012-06-27

**Authors:** Richard W. Francis, Katherine Thompson-Wicking, Kim W. Carter, Denise Anderson, Ursula R. Kees, Alex H. Beesley

**Affiliations:** 1 Division of Bioinformatics and Biostatistics, Telethon Institute for Child Health Research, Centre for Child Health Research, The University of Western Australia, Perth, Australia; 2 Division of Children’s Leukaemia and Cancer Research, Telethon Institute for Child Health Research, Centre for Child Health Research, The University of Western Australia, Perth, Australia; University of California, Los Angeles, United States of America

## Abstract

The hallmarks of many haematological malignancies and solid tumours are chromosomal translocations, which may lead to gene fusions. Recently, next-generation sequencing techniques at the transcriptome level (RNA-Seq) have been used to verify known and discover novel transcribed gene fusions. We present FusionFinder, a Perl-based software designed to automate the discovery of candidate gene fusion partners from single-end (SE) or paired-end (PE) RNA-Seq read data. FusionFinder was applied to data from a previously published analysis of the K562 chronic myeloid leukaemia (CML) cell line. Using FusionFinder we successfully replicated the findings of this study and detected additional previously unreported fusion genes in their dataset, which were confirmed experimentally. These included two isoforms of a fusion involving the genes *BRK1* and *VHL*, whose co-deletion has previously been associated with the prevalence and severity of renal-cell carcinoma. FusionFinder is made freely available for non-commercial use and can be downloaded from the project website (http://bioinformatics.childhealthresearch.org.au/software/fusionfinder/).

## Introduction

Translocations are rearrangements of regions of non-homologous chromosomes that can result in gene fusions as well as amplifications, deletions and inversions. The critical role of chromosomal abnormalities was recognised in the 1960s when Nowell and Hungerford identified the Philadelphia chromosome in CML [1]. This abnormality was later revealed to arise from a translocation between chromosomes 9 and 22, resulting in the *BCR-ABL* fusion gene [2], [3]. Typically associated with haematological malignancies, gene fusions have more recently been linked to solid tumours, including prostate, breast and lung cancers. According to the May 2012 release (v59) of the Catalogue Of Somatic Mutations In Cancer there are currently 7,732 fusions known to be associated with benign and malignant tumours [4] and many have been shown to play key roles in cancer initiation. Gene fusions can also be linked to clinical outcome, for example, the presence of the *BCR-ABL1* fusion is a powerful predictor of clinical outcome in paediatric acute lymphoblastic leukaemia. Furthermore, gene fusions provide ideal therapeutic targets since they create unique proteins not present in normal cells.

Systematic identification of gene fusions across cancer types is a major undertaking. Historically, the focus has been on molecular cytogenetic approaches, however, next-generation sequencing (NGS) technologies are increasingly being used, as they are considerably higher throughput and produce results at a much higher resolution than techniques such as fluorescence in-situ hybridization (FISH).

Transcriptome or RNA sequencing (RNA-Seq) [5] allows the analysis of several aspects of genome transcription, providing sequence data as well as the ability to detect alternative splicing events and quantify gene expression levels. Since genetic alterations identified by transcriptome sequencing are actively expressed there is greater confidence that they may directly contribute to the oncogenic phenotype rather than the many mutations identified from genome sequencing for which transcriptional status is not established. Further, as the transcriptome is considerably smaller than the genome, the fold-coverage from a typical sequencing run is much greater than for the whole genome providing a greater opportunity to identify mutations expressed even at very low levels. To date, transcriptome sequencing has been used to comprehensively characterise gene fusions in prostate, brain and breast cancer using both single-end (SE) [6], [7], [8] and paired-end (PE) [9] variants of the RNA-Seq technology.

A chromosomal breakpoint most commonly occurs within intronic or intergenic regions but can on rare occasions occur within exons [10], [11]. In either case, when performing RNA-Seq analysis, fusion transcript reads produced from sequencing translocation events will contain exonic sequence from two distinct genes. However, other biological mechanisms have been reported in the literature that cloud this simple concept, namely read-through transcription and trans-splicing. Read-through transcription occurs when the RNA-polymerase continues beyond the normal termination sequence and into an adjacent gene, usually within 20 kb [12]. Novel fusion transcripts are then generated by RNA-splicing from this larger pre-mRNA molecule. Trans-splicing is a process whereby exons from two independently processed transcripts from different locations in the genome (even from different chromosomes) become spliced together at the RNA level [13]. Complicating RNA-Seq analysis further, random chimeric transcripts can also occur at the RNA library preparation stage [14] when fragments of highly expressed transcripts become randomly attached to fragments from other genes. When RNA sequencing is performed, the resultant reads from any of these phenomena can appear to come from translocation-generated fusion transcripts as they too contain exonic sequence from two distinct genes.

There is some debate regarding the suitability of SE versus PE RNA-Seq approaches for the discovery of gene fusions. This is because, while more costly, PE sequencing can provide a greater depth of evidence for a gene fusion. With PE sequencing, reads are usually mapped separately to a normal transcriptome or genome reference to find pairs aligning to different genes, whilst with SE data, only those reads that directly span a fusion boundary are informative. However, recent advances in NGS platforms has resulted in vast increases in coverage and read length meaning that gene fusion boundaries are now adequately represented by SE reads even at low levels of expression. A number of studies have recently reported that the performance of SE sequencing is equal to [8], [15] and in some cases better than [16] PE, making SE sequencing a more cost effective approach to the discovery of gene fusions.

Previous work by Levin and colleagues [6] focussed on the discovery of fusion transcripts using “targeted RNA-Seq”, a technique involving a combination of SE RNA-Seq and hybridization capture methods. Using this method they enriched their initial sequencing data for 467 cancer-related genes in the CML cell line K562. Following mapping of sequencing reads to a reference transcriptome, candidate fusions were identified when the first 30 bases of the read matched a different gene to the last 30 bases.

In this project we have extended and fully automated this type of approach to develop the software FusionFinder. We applied our software to the SE Illumina data arising from the work by Levin and colleagues and, in addition to successfully replicating their findings, we have identified and experimentally confirmed novel fusion candidates expressed in K562 that may have functional relevance for the disease.

## Results

**Figure 1:**
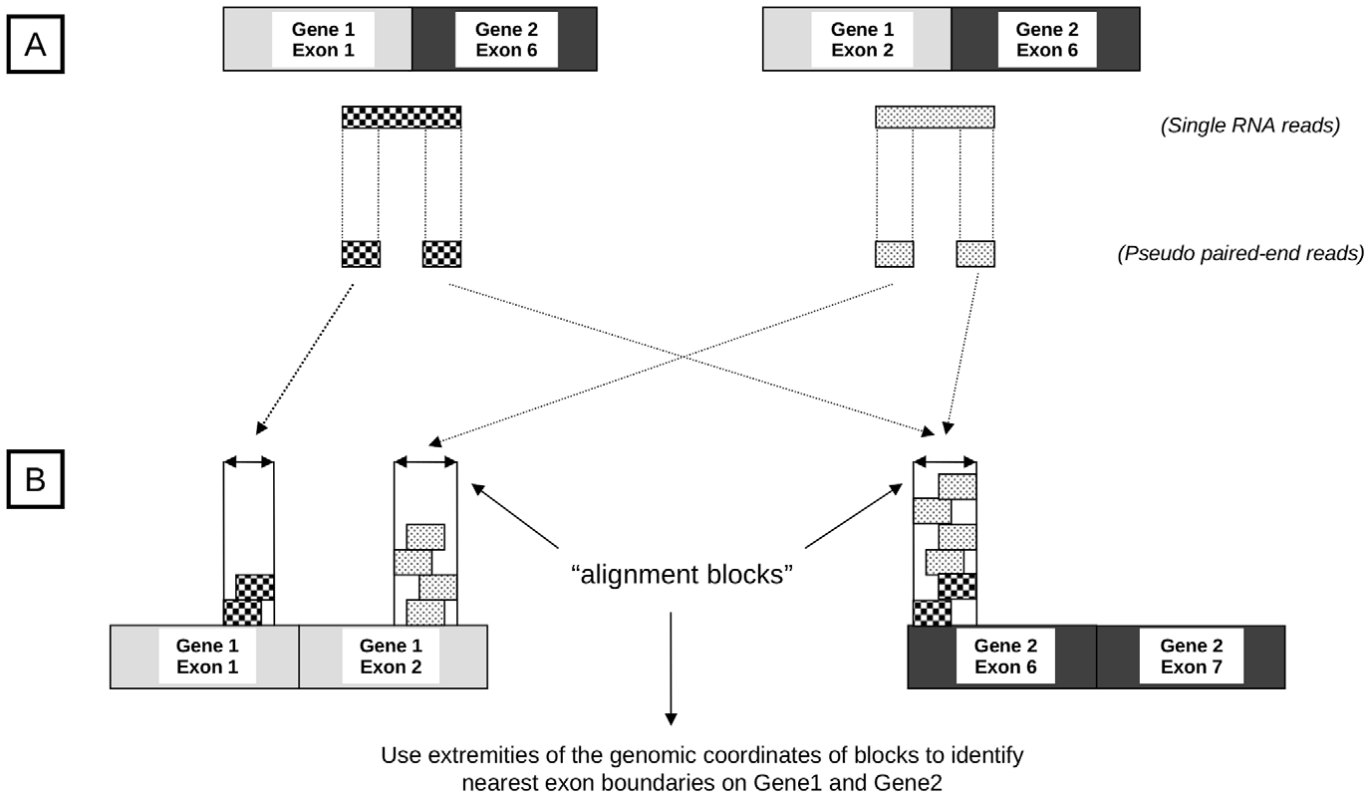
FusionFinder rationale. A) RNA-Seq produces millions of short reads, some of which will span the exon boundaries of hypothetical fusion transcripts between Gene 1 and Gene 2. Two different fusion isoforms involving different exons are shown, left and right, along with a single read that spans each breakpoint. Reads are split into smaller pseudo PE reads which can be aligned independently to a reference transcriptome. B) Alignment of pseudo PE reads against the reference transcriptome. One of each pair aligns to an exon on Gene 1 and the other aligns to an exon on Gene 2. Repeating this process for all other RNA-Seq reads creates “alignment blocks” from overlapping groups of aligned 5′ and 3′ pseudo PE reads and their genomic coordinates. Multiple alignment blocks on either gene (as for Gene 1 in the example) provide evidence for the existence of different isoforms of the fusion.

FusionFinder analyses FASTQ read data (of at least 50 nucleotides) to identify gene fusion candidates. This is achieved by performing the following integrated analysis and filtering steps, which are outlined in Box S1 and Figure 1. By default, all filters described are enabled but some can be disabled with command line options.

### Step 1. Alignment of Full Length Reads Against a Normal Coding Reference Transcriptome

The first step of the process (see Box S1) is to align the full length reads (e.g. 100 nucleotide reads in the case of Illumina HiSeq data) against a reference transcriptome containing only coding transcripts to identify those reads that fail to match to a normal dataset. We use Bowtie [17], to align the read data against this reference transcriptome and produce Sequence Alignment/Map (SAM) format [18] output, comprising one row per read where each read has either a single transcript hit or fails to match anything in the reference. The latter could be due to one of three main reasons: (i) the normal reference transcriptome is not comprehensive enough and does not contain the transcript to which this read should match (i.e. a completely novel but genuine non-fusion transcript or an expressed non-coding transcript); (ii) the read is of poor quality and the alignment software could not align it to any transcripts in the reference; (iii) the read overlaps the exon-exon junction of a fusion transcript, the sequence for which is understandably not in the reference transcriptome. It is these latter sequences that are the target for FusionFinder.

A reference coding transcriptome that is compatible with our analysis pipeline, can be obtained from our website [19], [20]. The references comprise all coding transcripts of all annotated genes within recent versions of Ensembl [21].

### Step 2. Creation of Pseudo Paired-end Reads

The next part of the process is to split each read with no matching hits from Step 1 into two smaller sections, so we can attempt to find sequences from different genes that match to each section (see Box S1 and Figure 1). From all full length reads having no hits in the reference transcriptome (i.e. reads that may potentially match a fusion gene, see Figure 1A), a pair of FASTQ “pseudo PE reads” are derived with each pair comprising the first n bases and the last n bases of the sequence of the full length read, where n represents a proportion (0.4 by default but no greater than 0.5) of the length of the full length read (Figure 1A). It is important to note that these pseudo PE reads retain the read ID of the parent full length read and are simply appended with ‘/1’ or ‘/2’ to distinguish each member of the pair (similar to PE reads), allowing them to be later reunited. In addition, a line graph of the overall quality of all full length reads is produced in this step.

### Step 3. Alignment of Pseudo PE Reads against the Coding Reference Transcriptome

The next step (see Box S1) is to align the pseudo PE reads against the reference transcriptome to establish which, if any, transcripts they align to. As in Step 1, we use Bowtie to align the pseudo PE reads independently against the coding reference transcriptome (Fig 1B). In Bowtie’s default mode, hits are determined based on 100% identity between a read and a transcript in the normal reference transcriptome over the first 28 bases of a read, while allowing for a 2 base mis-match. This acts as a quality control step for the data, filtering out poor quality reads.

### Step 4. Analysis and False-positive Filtering of the Pseudo PE Read Results

The next steps of the process perform several stages of analysis and filtering to narrow down the most likely candidate fusion transcripts using the available evidence (see Box S1). Initially each of the pseudo read pairs are reunited (based on their common ID) and examined for which, if any, transcripts they hit. The first two filters (Steps 4A & 4B) then discard data where the read pairs both match the same gene or where either read of the pair does not match anything in the reference transcriptome. If the read pairs hit different genes, which we’ve termed a G1:G2 (i.e. Gene 1:Gene 2) pair, the read pairs are stored in order to build up a body of evidence for the existence of this G1:G2 pair (example provided in Figure 1B). A G1:G2 pair can be regarded as a fusion candidate. The third and fourth filters (Steps 4C & 4D) remove false positive G1:G2 pairs where any read pairs hit either paralogous genes, or antisense transcripts that the alignment algorithm is unable to distinguish. These filters can be disabled using command line options if required. In the next filter (Step 4E) the read pairs are mapped back to the genome based on the coordinates where they aligned to G1 and G2 respectively. By default, Bowtie reports a single hit for a single read to a single transcript. Since transcript coordinates cannot be directly compared (due to differential exon usage between transcripts) all transcript/read alignment positions are transposed to genomic coordinates, which are then comparable. Using the canonical boundaries of each G1 or G2 exon, an assessment is made of whether the mapped positions are realistic given the size of the insert that should exist between them if the two exons were indeed fused in a transcript. For example two aligned 30mer pairs derived from a parent full length 76mer should have 16 bases between them when their respective mapped exon positions are assessed. Those read pairs mapping outside these constraints are filtered. The implementation of this last filter is responsible for eliminating many of the false positives including those arising from the existence of potential chimeric fusion artefacts in the RNA-Seq data. Finally (Step 4F) all pseudo PE reads evidencing all G1:G2 fusions are again realigned using Bowtie, firstly to the coding transcriptome reference and secondly to a reference containing only non-coding transcripts. In these alignment steps Bowtie is configured to allow all possible matches in each reference. G1:G2 pairs are filtered if their pseudo PE reads separately map to transcripts of the same gene in either reference. This step removes false positives arising as the result of genes sharing common exonic sequences. For example, the long intergenic non-coding (Linc) RNA *SUZ12P* is comprised of exons also found in both *LPHN1* and *SUZ12*. Similarly, many unrelated coding transcripts (some novel or un-annotated) contain common exons (e.g. *CCDC144A* and *USP32*). Without a multi-mapping read filter many of these examples would be incorrectly reported as being transcript fusions. The multi-mapping step is performed at this stage within FusionFinder (i.e. post-filtering, as opposed to during the initial alignments in Step 1) since the required computation time is significantly reduced with the smaller number of candidate reads.

### Step 5. Block Filtering and Identification of Fused Exons and Isoforms from Candidate Fusion Transcripts

This stage combines the remaining read evidence for each G1:G2 pair from *Step 4* to identify the exons involved in the fusion (see Box S1). Firstly (Step 5A) the genomic coordinates of each mapped read are combined to construct “alignment blocks” comprising multiple overlapping reads that map to the same area on G1 or G2 (see also Figure 1B). Each combination of blocks on G1 and G2 are then searched for overlapping repeat elements as indicated by RepeatMasker [22]. If the block on G1 overlaps the same repeat element class as the block on G2, the block combination is filtered out, as such reads are likely to represent false positives. This filter (Step 5B) is optional and can be disabled if required.

The genomic location of the extremities of “alignment blocks” are then used to retrieve the Ensembl exon closest to this location (Step 5C). As depicted in Figure 1B, these discrete blocks represent the exons involved in the fusion. A single block on each of G1 and G2 indicates a single isoform for this fusion. Multiple blocks indicate multiple exon involvement and the existence of different fusion isoforms consisting of different combinations of blocks from G1 and G2.

Next, the number of pseudo PE reads providing evidence for each G1:G2 pair following filtering is determined (Step 5D). Here the user can opt to filter G1:G2 pairs that are not evidenced by at least a minimum number of pseudo PE reads. Restricting results to higher numbers of reads reduces the likelihood of false positives, while the acceptance of smaller read numbers will capture those fusion transcripts expressed at lower levels.

Finally (Step 5E), categories are assigned to each G1:G2 pair based on the given evidence as follows:

1. Intrachromosomal - Genes originate from the same chromosome
2. Interchromosomal - Genes originate from different chromosomes
3. Potential Readthrough - Genes on the same chromosome and strand and < = 20 kb apart
4. Inversion - Genes on the same chromosome but different strands

### Output Files

Four main output files are produced. The first is a summary file, which contains a ranked list of fusion candidates based on their evidence strength (total number of sequence reads). The file provides the Ensembl and HUGO (Human Genome Organization) Gene Nomenclature Committee (HGNC) common name identifiers for G1 and G2, the number of blocks on each gene, an indication of how many isoforms exist for each G1:G2 pair and the category of fusion indicated by the pair. The second file gives the full details for each isoform of G1 and G2 and includes the genomic coordinates of the alignment blocks on G1 and G2, and their respective corresponding Ensembl exon IDs. For each isoform of a candidate fusion, the remaining two files provide (i) the sequences of the pseudo PE reads and the corresponding full length parent read and (ii) a forward three-frame translation of the fused nucleotide sequence of the two exons implicated in the candidate fusion. While FusionFinder is running, the software outputs all filtered read data to a separate file that contains a flag denoting the stage at which each read was filtered. In addition, statistics are produced detailing the raw numbers of reads that have been filtered at various stages of the algorithm and those remaining that provide the evidence for the fusion candidates contained in the summary file.

### Further Investigation of Fusion Candidates

**Figure 2:**
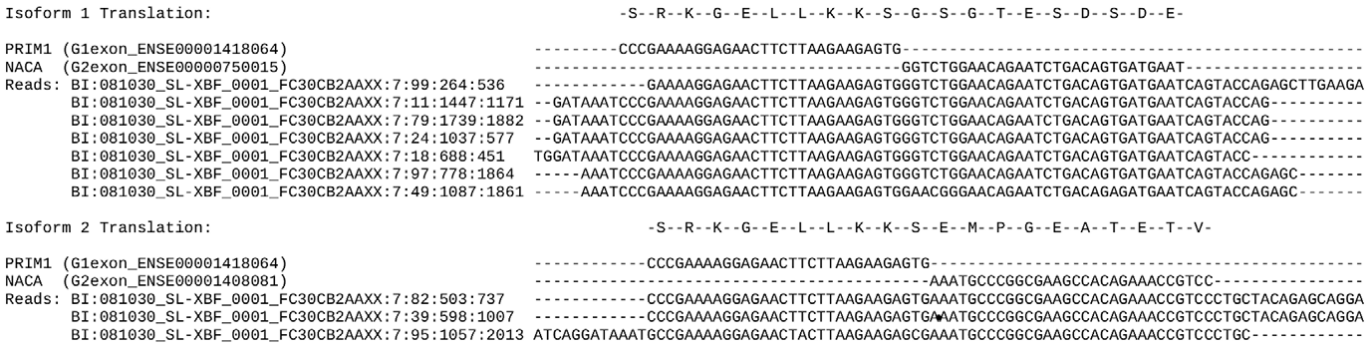
Identification of the transcript breakpoint in each *PRIM1*:*NACA* isoform. Alignments of the full length 76mer reads providing evidence for the two isoforms of *PRIM1*:*NACA* (i.e. as originally identified by Levin et al, top, and the novel isoform identified by FusionFinder, bottom) against the last 30 bases of the implicated *PRIM1* (G1) exon and the first 30 bases of the *NACA* (G2) exon. The transcript breakpoint can be clearly seen where the *PRIM1* exon ends and the *NACA* exon begins. Also displayed is an in-frame translation of the G1 exon from wild type *PRIM1*, running into the fused *NACA* exon. Both isoforms retain an open reading frame despite different exon usage.

Using the common names of a G1:G2 pair of interest one can use FusionFinder to generate alignments to assist in the experimental confirmation of the candidate by RT-PCR. These alignments detail a) where the pseudo PE reads align to G1 and G2 and b) the exact location of the transcript breakpoint on the aligned parent reads (an example of this alignment is given in Figure 2).

### Software Implementation

FusionFinder has been tested on various versions of Perl from 5.10 onwards and makes extensive use of the BioPerl [23] and Ensembl API [21] libraries, which are required to be installed locally with the software. Several other Perl libraries are also required (standard libraries available in the Comprehensive Perl Archive Network) that are detailed further in the software manual available from the project website [19], [20]. We have comprehensively tested FusionFinder on 64-bit Linux, but it can be run on both Windows and MacOS platforms provided Perl and the aforementioned dependencies are installed. FusionFinder requires access to an Ensembl database (ideally a locally installed) to perform some sections of the identification process.

The commands to run FusionFinder are described in the online user manual available from the project website [19], [20]. The source code for FusionFinder is made freely available from our website under the GNU General Public License (GPL).

### Application of FusionFinder to a Published RNA-Seq Dataset

We applied FusionFinder to data arising from work by Levin and colleagues [6] who performed a targeted RNA-Seq analysis of the K562 CML cell line. They generated sequencing data both pre- and post-enrichment for 467 cancer-related genes. We used the dataset representing the sequencing produced post-enrichment, which consisted of 14 million 76mer SE reads in FASTQ format. We refer to this dataset hereon as “the Levin dataset”. We generated 30mer pseudo PE reads and searched for candidates that were evidenced by ≥4 pseudo PE reads. We used a coding transcriptome reference containing only those Ensembl transcripts with a known translation and source data from a local installation of Ensembl (release 62 - April 2011). All read alignments were performed with Bowtie (version 0.12.7), allowing for a 2 base mismatch.

Our analysis produced a final list of 9 fusion candidates. Table 1 gives the details of these candidates as presented in the FusionFinder summary file. Table 2 shows the details found in the FusionFinder isoforms file for each of the fusion candidates from Table 1 that were previously reported by Levin *et al*.

**Table 1:** Summary file showing the 9 candidates from the FusionFinder analysis of the Levin dataset.

Index Number	G1_Ensembl_HGNC_ID	G1_chromosome	G2_Ensembl_HGNC_ID	G2_chromosome	totalreads	G1_blocks	G2_blocks	isoforms	category
1	ENSG00000186716 (BCR)	chromosome_22	ENSG00000097007 (ABL1)	chromosome_9	425	1	1	1	INTERCHROMOSOMAL
2	ENSG00000126883 (NUP214)	chromosome_9	ENSG00000172967 (XKR3)	chromosome_22	78	1	3	3	INTERCHROMOSOMAL
3	ENSG00000198056 (PRIM1)	chromosome_12	ENSG00000196531 (NACA)	chromosome_12	10	1	2	2	POTENTIAL_READTHROUGH, INTRACHROMOSOMAL
4	ENSG00000180198 (RCC1)	chromosome_1	ENSG00000073921 (PICALM)	chromosome_11	10	2	3	4	INTERCHROMOSOMAL
5	ENSG00000112759 (SLC29A1)	chromosome_6	ENSG00000096384 (HSP90AB1)	chromosome_6	9	1	1	1	POTENTIAL_READTHROUGH, INTRACHROMOSOMAL
6	ENSG00000143702 (CEP170)	chromosome_1	ENSG00000182185 (RAD51L1)	chromosome_14	7	1	1	1	INTERCHROMOSOMAL
7	ENSG00000213672 (NCKIPSD)	chromosome_3	ENSG00000008300 (CELSR3)	chromosome_3	6	1	1	1	POTENTIAL_READTHROUGH, INTRACHROMOSOMAL
8	ENSG00000254999 (C3orf10)	chromosome_3	ENSG00000134086 (VHL)	chromosome_3	5	2	2	2	INTRACHROMOSOMAL
9	ENSG00000110455 (ACCS)	chromosome_11	ENSG00000151348 (EXT2)	chromosome_11	4	3	1	3	POTENTIAL_READTHROUGH, INTRACHROMOSOMAL

Displayed are the Ensembl gene IDs and HGNC IDs of the G1:G2 pair, their chromosome number, the total number of read pairs providing evidence for the G1:G2 pair in question (totalreads), the number of aligned blocks on each gene (G1_blocks/G2_blocks) the number of potential isoforms identified and the assigned category of each candidate. The index number is used to refer to particular candidates in the main article. Candidates numbered 1–5 and 7 were previously reported by Levin *et al*.

**Table 2:** FusionFinder isoforms file for the six common fusion candidates reported by both FusionFinder and Levin *et al*.

G1_Ensembl_HGNC_ID	G1_chromosome	G2_Ensembl_HGNC_ID	G2_chromosome	isoreads	totalreads	G1_block	G1_exon	G1_expos	G1_str	G2_block	G2_exon	G2_expos	G2_str
ENSG00000186716 (BCR)	chromosome_22	ENSG00000097007 (ABL1)	chromosome_9	425	425	23632551–23632600	ENSE00001781765	0	1	133729451–133729500	ENSE00002219347	0	1
ENSG00000126883 (NUP214)	chromosome_9	ENSG00000172967 (XKR3)	chromosome_22	2	78	134074371–134074400	ENSE00002297135	2	1	17280871–17280900	ENSE00001196508	14	-1
ENSG00000126883 (NUP214)	chromosome_9	ENSG00000172967 (XKR3)	chromosome_22	67	78	134074352–134074402	ENSE00002297135	0	1	17288926–17288973	ENSE00001303813	0	-1
ENSG00000126883 (NUP214)	chromosome_9	ENSG00000172967 (XKR3)	chromosome_22	9	78	134074370–134074399	ENSE00002297135	3	1	17265257–17265286	ENSE00001305313	13	-1
ENSG00000112759 (SLC29A1)	chromosome_6	ENSG00000096384 (HSP90AB1)	chromosome_6	9	9	44200122–44200165	ENSE00001140400	0	1	44216369–44216415	ENSE00002305523	2	1
ENSG00000180198 (RCC1)	chromosome_1	ENSG00000073921 (PICALM)	chromosome_11	2	10	28834640–28834672	ENSE00002271332	0	1	85694971–85695007	ENSE00000989277	9	-1
ENSG00000180198 (RCC1)	chromosome_1	ENSG00000073921 (PICALM)	chromosome_11	2	10	28832567–28832596	ENSE00001461695	0	1	85694971–85695000	ENSE00000989277	16	-1
ENSG00000180198 (RCC1)	chromosome_1	ENSG00000073921 (PICALM)	chromosome_11	5	10	28834640–28834659	ENSE00002271332	13	1	85718594–85718623	ENSE00000989273	3	-1
ENSG00000180198 (RCC1)	chromosome_1	ENSG00000073921 (PICALM)	chromosome_11	1	10	28834640–28834654	ENSE00002271332	18	1	85693004–85693031	ENSE00002191427	0	-1
ENSG00000198056 (PRIM1)	chromosome_12	ENSG00000196531 (NACA)	chromosome_12	7	10	57127931–57127969	ENSE00001418064	0	-1	57108423–57108464	ENSE00000750015	7	-1
ENSG00000198056 (PRIM1)	chromosome_12	ENSG00000196531 (NACA)	chromosome_12	3	10	57127931–57127972	ENSE00001418064	0	-1	57118262–57118303	ENSE00001408081	4	-1
ENSG00000213672 (NCKIPSD)	chromosome_3	ENSG00000008300 (CELSR3)	chromosome_3	6	6	48716003–48716045	ENSE00001204809	6	-1	48694742–48694781	ENSE00001170666	0	-1

Displayed are the Ensembl gene IDs and HGNC IDs of the G1:G2 pair, their chromosome number, the number of read pairs providing evidence for the particular isoform (isoreads), the total number of read pairs providing evidence for the G1:G2 pair in question (totalreads), the genomic coordinates for the aligned block on G1 or G2 (G1_block, G2_block respectively) the corresponding closest Ensembl exon to the aligned block (G1_exon/G2_exon) the proximity (distance in bp) of the aligned block to the end or start of the corresponding exon (G1_expos, G2_expos respectively) and the strand where each gene of the pair is located (G1_str, G2_str respectively). Note the novel second isoform of the *PRIM1:NACA* fusion discovered in the current analysis.

### Replication of the Levin Results

Using a similar (but not fully automated) technique to that presented here, Levin and colleagues confirmed the findings of Maher *et al* [9], who also analysed the K562 cell line using a PE sequencing approach and observed the fusions *BCR:ABL1* and *NUP214:XKR3*, fusions 1 and 2 in Table 1. In addition to these, when analysing the enriched dataset, Levin *et al*. reported the previously unobserved fusion transcripts listed at 3, 4, 5 and 7 in Table 1. Overall they reported four isoforms of the *NUP214:XKR3* fusion (involving specific combinations of exons 29 and 27 of *NUP214* and exons 2, 3 and 4 of *XKR3)* and four isoforms of the *RCC1:PICALM* fusion.

Within our candidates, we observed all six fusion events reported by Levin and colleagues (highlighted in Table 1). These included the three isoforms of *NUP214:XKR3* involving exon 29 of *NUP214* and all four isoforms of *RCC1:PICALM*. The fourth isoform of *NUP214:XKR3* involving exon 27 of *NUP214* was found in a separate analysis using a value of 0.2 when generating the 3' pseudo PE reads (data not shown). Importantly, FusionFinder also reported that due to their genomic proximity, candidates listed as 3, 5 and 7 in Table 1 are potential read-through transcripts as described elsewhere [16].

### Additional Findings

In addition to those fusions found by Levin *et al*., FusionFinder generated evidence for three other gene fusions expressed in the K562 cell line (Table 1, non-shaded) as well as a second isoform of the *PRIM1:NACA* fusion (see Table 2). These new candidates are *ACCS:EXT2*, *C3orf10:VHL* and *CEP170:RAD51L1* the first two each having multiple associated isoforms (Table 1).

Figure 2 shows the alignment for both identified isoforms of *PRIM1:NACA* (i.e. the original isoform identified by Levin *et al*., and the novel isoform identified by FusionFinder) and an in-frame translation for the region 30 bases upstream and downstream of the transcript breakpoint. In wild type *PRIM1*, a stop codon lies within the exon downstream of the implicated transcript breakpoint. However, in both fusion isoforms an open reading frame is retained through the fused *NACA* exon, therefore generating a sequence coding for a novel fusion peptide.

### Experimental Confirmation of Novel Fusions

**Figure 3:**
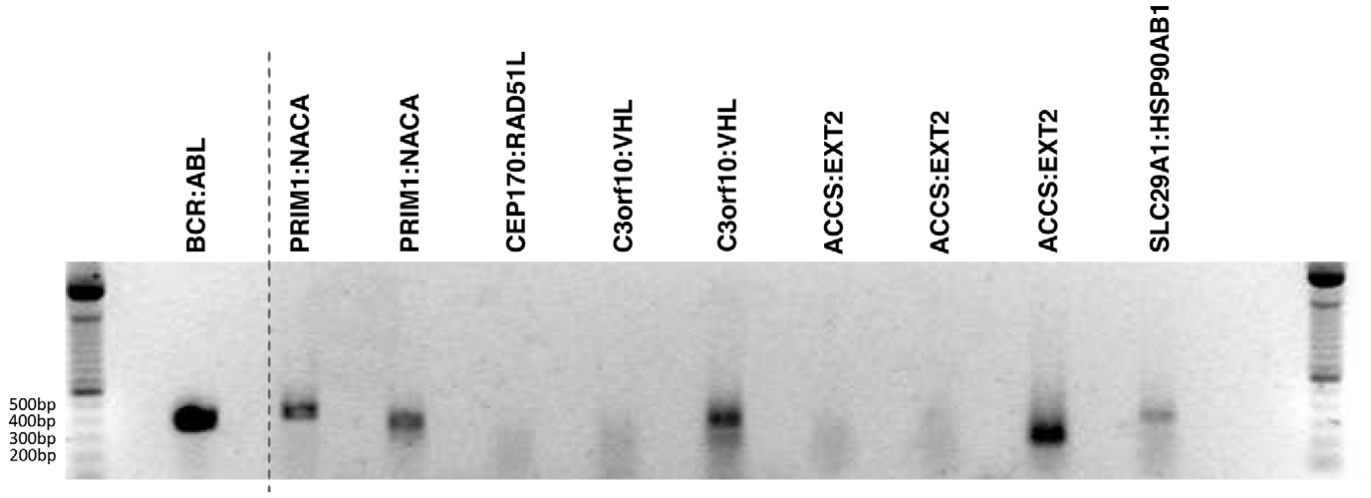
RT-PCR validation of the fusion candidates. Primers were designed around the individual fusion breakpoints and cDNA was synthesised using gene-specific primers. Products were successfully amplified for the following fusion isoforms; *BCR:ABL* (380 bp, lane 1), *PRIM1:NACA* isoform 1 (400 bp, lane 2), *PRIM1:NACA* isoform 2 (340 bp, lane 3), *C3orf10:VHL* isoform 2 (340 bp, lane 6), *ACCS:EXT2* isoform 3 (230 bp, lane 9) and *SLC29A1:HSP90AB1* (340 bp, lane 10). No product could be amplified from *CEP170:RAD51L1* (lane 4), *C3orf10:VHL* isoform 1 (lane 5), *ACCS:EXT2* isoform 1 or *ACCS:EXT* isoform 2 (lanes 7 and 8). The corresponding negative controls for each reaction are in the lanes proceeding each reaction. All detected fusion products were validated by Sanger sequencing.

Using RT-PCR and Sanger sequencing we experimentally confirmed the existence of two of the three novel fusions (8 and 9 in Table 1) as well as the novel isoform of *PRIM1:NACA* using RNA extracted from the commercially available K562 cell line (Figure 3). In addition we confirmed that the sequence at the transcript breakpoints of the previously identified fusions *BCR:ABL1* and *SLC29A1:HSP90AB1* were correctly predicted by FusionFinder. At least one predicted isoform of each confirmed novel fusion was detectable in our particular K562 cell line. Failure to detect every isoform of each predicted gene fusion is attributable to fact that we did not employ the targeted-enrichment protocol used by Levin *et al* prior to experimental confirmation, which would dramatically increase the representation of these transcripts within the RNA pool.

### Comparison to Existing Software on Real and Simulated Data

Recently published software for the analysis of RNA-Seq data for gene fusions include FusionMap [16], FusionSeq [24], FusionHunter [25], deFuse [26] and Tophat-Fusion [15]. Of these, both FusionMap and Tophat-Fusion can process SE read data. FusionMap uses a similar strategy to FusionFinder by splitting reads into smaller sections and finding fusion candidates where sections align to different genes on an annotated genomic reference prior to filtering. Tophat-Fusion uses to a two stage process of firstly aligning reads with a modified version of the spliced alignment software Tophat [27] to a genomic reference before secondly performing a post processing step to overlay annotation and perform filtering.

To compare the performance of FusionFinder with FusionMap and Tophat-Fusion we have run a full analysis with all three tools using the Levin dataset. For this dataset, comprising 14 million 76mer reads, a complete analysis with FusionFinder took approximately 3.1 hours on a single 2.4 GHz core of a multi-core AMD server with a peak memory usage of 1.8GB and using data from a local Ensembl (release 62) mirror. FusionMap (version 2012-03-03) with comparable parameters (α = 25, β = 1, G = 2) and preloaded reference data running the same analysis using Mono (version 2.10.8) under 64-bit linux, again on a single 2.4 GHz core, took 2.1 hours to complete and at its peak consumed 7.2 GB memory. Tophat-Fusion with comparable parameters (alignment phase: –fusion-min-dist 10000 and post-processing: –num-fusion-reads 4–num-fusion-pairs 0–num-fusion-both 4) and reference data on the same platform took 15 hours to complete and consumed 9.6 GB memory at its peak during the post-processing step. Although the run time for FusionFinder is slightly slower than FusionMap on a single core both are considerably faster than Tophat-Fusion. In addition FusionFinder consumes far less memory under a Linux operating system than both FusionMap and Tophat-Fusion. It should be noted that both FusionMap and Tophat-Fusion can be run on multiple CPU cores and with the same dataset and parameters but with 5 CPU cores, the analysis took 0.8 hours and 4.5 hours respectively. Similarly Bowtie can be run on multiple CPU cores and using 5 cores for the alignment steps in the FusionFinder protocol improves the total analysis time to 2.4 hours. We are currently working on a fully multithreaded version of FusionFinder. These data are summarised in Table 3 and a detailed breakdown of resources used by FusionFinder at each step of the protocol can be found in Table S4.

**Table 3:** Performance comparison of FusionFinder, FusionMap and Tophat-Fusion in an analysis of the Levin dataset.

	FusionFinder	FusionMap	Tophat-Fusion
**Total time taken - single core (hrs)**	3.1	2.1	15.0
**Total time taken - 5 cores (hrs)**	2.4	0.8	4.5
**Peak Memory - single core (GB)**	1.8	7.2	9.6

Data based on the analysis of the Levin dataset comprising 14 million 76 mer reads, using either a single 2.4 GHz core or 5 cores of a 64-bit linux machine with multiple AMD Opteron 8431 CPUs and 32GB memory. The parameters used for each analysis are in the main text and the raw results for each analysis can be found in Table 1 and Tables S1 a and b.

In line with previous reports [16] our analysis of the Levin dataset with FusionMap confirmed the findings of Levin *et al*. and reported an additional 57 candidates in this dataset. In comparison, Tophat-Fusion reported 12 candidate fusions but did not successfully identify all those reported by Levin *et al* or the additional candidates reported by FusionFinder, even when we allowed for the detection of the read-through transcripts we observed. Tophat-Fusion only reported two of the three isoforms of *NUP214:XKR3* reported by FusionFinder and did not report *CEP170:RAD51L1* but did report an additional isoform of *BCR:ABL1* which was not reported by FusionFinder or FusionMap. The results of these analyses are presented in Table S1 a and b.

**Figure 4:**
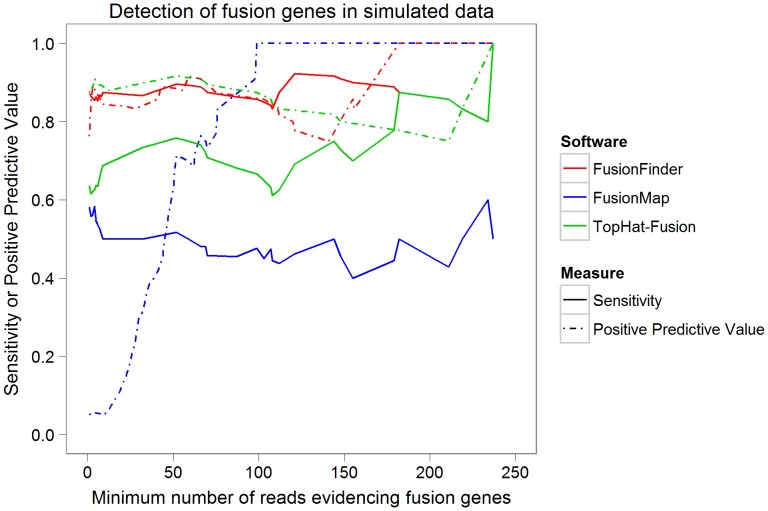
Comparison of sensitivity and PPV for FusionFinder, FusionMap and Tophat-Fusion. To compare the sensitivity and PPV of FusionFinder, FusionMap and Tophat-Fusion to detect fusion genes, each software was used to analyse a randomly generated dataset simulating normal genes and 55 fusion genes. Calculations of sensitivity and PPV were made for subgroups of the results based on the number of reads evidencing the fusion genes predicted by each software. FusionFinder shows consistently higher sensitivity than both FusionMap and Tophat-Fusion and shows a generally higher PPV than FusionMap and similar PPV to Tophat-Fusion.

To further compare the performance of each software we generated a simulated dataset of approximately 13.5 million SE 75 nucleotide reads (see Methods). The dataset contained normal background reads and “fusion reads” representing the transcript breakpoint of 55 fusion transcripts generated at random (see Table S2a). The dataset simulated fusions designed to represent both high and low levels of expression with read numbers per fusion transcript ranging from 1 – 295. We ran FusionFinder, FusionMap and Tophat-Fusion against this dataset. FusionFinder was run with default parameters, generating 30mer pseudo PE reads. FusionMap was run with comparable parameters (α = 30, β = 1, G = 2, MinimalHit = 1), though we note that in order for FusionMap to detect any simulated fusions it was necessary to alter the MinimalRescuedReadNumber parameter to 0. Tophat-Fusion was also run using comparable parameters for the post-processing step of the protocol (–num-fusion-reads 1–num-fusion-pairs 0–num-fusion-both 1). Sensitivity and Positive Predictive Values (PPV) were then calculated for the simulated dataset to assess the ability of each software to accurately detect simulated fusion genes (see Methods). Table 4 summarises the overall results of this analysis whilst a plot of these data is shown in Figure 4. The raw results from each software can be found in Tables S2 b, c and d. The performance measures were calculated on subgroups of fusion genes where subgroups were selected based on the number of reads (*i*) evidencing the fusion genes predicted by each software. For example, the point marked at 100 on the x axis of Figure 4 shows performance measures for all predicted fusion genes that were found to be evidenced by 100 or more reads.

**Table 4:** Summary of the overall comparative performance of FusionFinder, FusionMap and Tophat-Fusion on a simulated dataset.

	Sensitivity	PPV	True positive fusions	True positivereads	False positive fusions	False positive reads
**FusionFinder**	0.87	0.76	48	2937	15	433
**FusionMap**	0.58	0.05	32	2065	582	7045
**Tophat-Fusion**	0.64	0.88	35	3120	5	446

A total of 55 fusion genes were simulated. Sensitivity and PPV measures were compiled from predicted fusion genes evidenced by 1 read or more (i.e. all data). True positive fusions/reads relate to the accurate prediction of simulated fusions whereas false positive fusions/reads relate to all other predictions including synonymous fusions. For FusionFinder, all false positive genes and consequently all false positive reads were from synonymous fusions (see Table S2b). FusionFinder detects more simulated fusions and significantly fewer false positives than FusionMap with consistently greater sensitivity and PPV. FusionFinder showed a higher sensitivity and comparable PPV to Tophat-Fusion.

Among the fusion gene predictions made by each software are what we have termed “synonymous fusions”. These are where at least one of the identified gene partners has been inaccurately predicted because it shares high sequence similarity with the expected partner gene, possibly because it is a member of the same gene family (for example, an *S100A3*:*SULT1A4* fusion may be detected as an *S100A3*:*SULT1A3* fusion). Although the informed researcher would frequently be able to distinguish these fusions by visual comparison with other candidates in the output files, in our assessment of sensitivity and PPV such synonymous fusions were considered to be false positives to provide the most stringent assessment of each software.

It can be seen from Figure 4 and Table 4 that FusionFinder shows greater sensitivity and generally greater PPV than FusionMap in the detection of our simulated fusion genes. Figure 4 also shows that FusionFinder compares favourably against Tophat-Fusion with an overall greater sensitivity and a comparable PPV. With regard to the overall sensitivity in Table 4, FusionFinder detected 87% of the 55 simulated fusion genes, FusionMap reported 58% and Tophat-Fusion reported 64%. Importantly, FusionFinder and Tophat-Fusion only detected 15 and 5 false positives respectively (some of which were synonymous fusions - see Table S2 b, c and d) giving them a comparable PPV. In contrast FusionMap reported 582 false positives, which represents 95% of the returned results respectively (Table 4). This has a considerable effect on the PPV in Figure 4 at low read levels with FusionMap remaining significantly lower than both FusionFinder and Tophat-Fusion. Consequently, in the case of FusionMap the user is returned a large list of potential fusion genes consisting primarily of false positives. In contrast, the candidates reported by FusionFinder will be more robust and more likely to be confirmed experimentally.

For two of the fifty-five simulated fusions, the partner genes contained repeats of the same class at the transcript breakpoint. These gene fusions were detected by FusionFinder but due to the RepeatMask filter, were subsequently filtered. However, both of these appeared in the FusionMap results, suggesting that FusionMap does not take the presence of repeats in to account. This could explain the occurrence of so many false positives in FusionMap’s results. Both of these simulated fusions were also filtered by Tophat-Fusion.

It should be noted that when both FusionMap and Tophat-Fusion detected a simulated fusion gene they consistently detected all simulated fusion reads, however although FusionFinder detected more simulated fusion genes it did not consistently detect all fusion reads. This is because, FusionFinder analyses data from an alignment using Bowtie’s default parameters which does not provide results for multi-mapping reads. This means that given two genes from the same family, sharing high sequence identity, a read has an equal chance of hitting either of these genes. As a result, the expected fusion reads are distributed between all synonymous fusions. We are working on a method to analyse alignments of multi-mapping reads, which will significantly increase the numbers of reads detected.

### Application of FusionFinder to a PE Dataset

While FusionFinder is more suited to the analysis of SE data, it can also be used to analyse PE data, by considering each PE reads file as a separate SE reads file. We applied FusionFinder to PE RNA-Seq data from the MCF-7 breast cancer cell line as previously described [28] and also analysed by the authors of Tophat-Fusion [15]. This dataset comprises approximately 17 million 50 bp PE reads. Table 5 shows the results of an analysis of this data with FusionFinder using default parameters but creating 25 bp pseudo PE reads and searching for candidates with at least two reads evidencing them. We found seven fusions, six of which have been previously reported [28], [29]. Only three of these fusions were found by Tophat-Fusion [15].

**Table 5:** Summary file showing the 7 candidates from the FusionFinder analysis of the MCF-7 Breast Cancer cell line paired-end dataset.

Index Number	G1_Ensembl_HGNC_ID	G1_chromosome	G2_Ensembl_HGNC_ID	G2_chromosome	totalreads	G1_blocks	G2_blocks	isoforms	category
1	ENSG00000124243 (BCAS4)	chromosome_20	ENSG00000141376 (BCAS3)	chromosome_17	18	1	1	1	INTERCHROMOSOMAL
2	ENSG00000124198 (ARFGEF2)	chromosome_20	ENSG00000196562 (SULF2)	chromosome_20	6	1	1	1	INVERSION,INTRACHROMOSOMAL
3	ENSG00000108443 (RPS6KB1)	chromosome_17	ENSG00000062716 (TMEM49)	chromosome_17	5	1	1	1	INTRACHROMOSOMAL
4	ENSG00000086712 (TXLNG)	chromosome_X	ENSG00000169895 (SYAP1)	chromosome_X	3	1	1	1	INTRACHROMOSOMAL
5	ENSG00000127616 (SMARCA4)	chromosome_19	ENSG00000142453 (CARM1)	chromosome_19	2	1	1	1	INTRACHROMOSOMAL
6	ENSG00000163399 (ATP1A1)	chromosome_1	ENSG00000020256 (ZFP64)	chromosome_20	2	1	1	1	INTERCHROMOSOMAL
7	ENSG00000175029 (CTBP2)	chromosome_10	ENSG00000038532 (CLEC16A)	chromosome_16	2	1	1	1	INTERCHROMOSOMAL

Displayed are the Ensembl gene IDs and HGNC IDs of the G1:G2 pair, their chromosome number, the total number of read pairs providing evidence for the G1:G2 pair in question (totalreads), the number of aligned blocks on each gene (G1_blocks/G2_blocks) the number of potential isoforms identified and the assigned category of each candidate. Candidates numbered 1–6 have previously been reported.

## Discussion

FusionFinder is a Perl based software suite designed for the discovery of fusion transcripts and their isoforms. We have demonstrated the use of FusionFinder by applying it to publicly available SE (the Levin dataset) and PE (MCF-7) data. In the case of the Levin dataset we successfully replicated previously published findings (including the discovery of an additional isoform of the previously reported *PRIM1:NACA* fusion, Figure 2) and revealed three other candidates for further study. In total there were 9 fusion candidates passing all filters. The Levin dataset represents a targeted RNA-Seq approach wherein 467 cancer-related genes were representationally enriched. As expected, at least one or both of the genes implicated in each of our fusion candidates were enriched for by Levin *et al*. Aside from the six fusion transcripts reported by Levin *et al*., the three additional fusions we have identified with FusionFinder are also of biological interest:

*C3orf10:VHL* or *BRK1:VHL* (Table 1, #8) - *VHL* is a tumour suppressor gene deleted in von Hippel Lindau disease, an autosomal dominant familial cancer syndrome that can give rise to pheochromocytoma and tumours of the kidney, central nervous system, pancreas, retina and epididymis. The *BRK1* gene (*BRICK1*, *C3orf10* or *HSPC300*) lies upstream of *VHL* on chromsome 17 and is involved in the branched nucleation of actin fibres. Although no fusions of the two genes have to date been reported, co-deletion of *BRK1* has been shown to alter the prevalence and severity of renal-cell carcinoma in patients with *VHL* deletion [30], [31]. Such co-deletion has been attributed to *Alu*-mediated recombination since the genes lie in a region of high *Alu* density [30] and it is likely that gene fusion could occur by a similar mechanism with deletion of the intergenic region. This is consistent with the transcript breakpoint in isoform 2, which appears to involve an intra-exonic break in the *C3orf10* gene (sequences available in File S1), arguing against an RNA-splicing mechanism. The oncogenic effect of a fusion between these genes is not known but their co-operating role in the development of renal cell carcinoma suggests that the existence of this fusion transcript in the K562 CML cell line is likely to be functionally relevant. The expression of one of the isoforms of this fusion was confirmed by RT-PCR in our K562 cell line.

*ACCS:EXT2* (Table 1, #9) - *EXT2* is a tumour supressor gene implicated in multiple osteochondroma [32], with the gene being affected by a wide-range of mutations including frameshift and splice-site mutations. The proximity of the *ACCS* and *EXT2* genes suggests that this potential fusion may result from read-through transcription and subsequent RNA splicing, similar to the situation with *PRIM1:NACA* (Table 1, #4) and a number of others from Table 1. Again, expression of this gene fusion was confirmed by RT-PCR in our K562 cell line.

*CEP170:RAD51L1* (Table 1, #6) – *RAD51L1* is a DNA repair gene, and is a known translocation partner in a series of benign solid tumours [33]. Although this fusion was not confirmed experimentally in our K562 cell line, this is likely due to the fact that we did not perform the enrichment protocol employed by Levin and colleagues prior to experimental confirmation.

The results of our software comparison clearly demonstrate the utility of FusionFinder versus two other existing methods. As well as more consistently predicting gene fusions, FusionFinder provides more detailed sequence based output to assist in the experimental confirmation of fusion candidates. Using the three frame translation of the area around the predicted transcript breakpoint one can quickly establish whether an open reading frame exists across the fused exons. Furthermore, the alignments produced by FusionFinder provide a more detailed picture of the context of the fusion, which is valuable information when seeking experimental confirmation. In addition to demonstrating that FusionFinder can detect gene fusions in enriched SE data (Levin dataset) and simulated SE data we have also shown the detection of gene fusions in publicly available PE data (MCF-7). Furthermore we have applied FusionFinder to our own in-house RNA-Seq data and have characterised a novel fusion transcript expressed in a rare paediatric carcinoma (manuscript under review).

A few assumptions exist that must be considered when using FusionFinder, although these would apply to most software of this nature aimed at analysing RNA-Seq data. Firstly, FusionFinder will only detect transcribed gene fusions because RNA-Seq only sequences at the transcript level. Secondly, RNA-Seq is a quantitative technology and transcripts that are more highly expressed are sequenced at higher coverage, meaning that transcripts expressed at lower levels will be harder to detect. Despite this caveat, those that are represented at low levels, even down to a single read, will be pulled out of the sea of false positives by the extensive logical filtering within FusionFinder. Thirdly, FusionFinder cannot distinguish between a physical fusion, RNA trans-splicing or read-through transcription. Fourthly, FusionFinder is designed to detect fusions occurring at canonical exon junctions. Therefore it will not typically detect fusions involving intra-exon breakpoints, which are relatively rare [10], [11]. However it should be noted that FusionFinder was able to detect the experimentally confirmed *C3orf10*:*VHL* (isoform 2) fusion, alignments for which implicate an intra-exon break. An explanation for this particular case can be found in File S1. Finally, it is essential that the transcriptome reference library is comprehensive enough to reliably capture all known transcripts. The reference libraries we provide comprise annotated transcripts contained within Ensembl and are updated in line with new builds of Ensembl. Using reference data from Ensembl has an additional advantage in that when a new Ensembl build is released it is a very straight forward process for the user to download a new reference dataset from our website [19], [20], update their Ensembl API and point to the new build. In doing so the user always has access to the most current reference annotation.

The candidates identified by FusionFinder are predictions based on sequence evidence and laboratory confirmation is required to determine whether or not a transcribed fusion gene will be functional. The alignments generated by FusionFinder assist in this process by providing the sequence of the fusion candidate at the point of the transcript breakpoint and also provide details regarding whether or not this sequence retains an open reading frame. Once the sequence of the fused exons is known, it is possible to generate predicted models of the full fusion transcript and subsequently assess expression levels using the algorithms provided within software such as Cufflinks [34] and EdgeR [35]. Whilst FusionFinder identifies any expressed fusion transcripts from the sample under investigation, researchers must of course consider cellular heterogeneity in their interpretation of the data, and the possibility that signals may arise from multiple clones (or different cell types) within the specimen.

As next-generation sequencing technologies become more affordable, so the number of studies using this technology to discover fusion transcripts will increase. There is a great need for adequate analysis software that can rapidly interrogate such large amounts of data to reveal the patterns therein. FusionFinder provides a logical and flexible software solution to this end that will facilitate the automated discovery of fusion transcripts in RNA-Seq data. FusionFinder has not only validated the fusion transcripts previously reported for the K562 CML cell line but has also identified novel candidate fusions. Of these, *BRK1*:*VHL* represents the fusion of two genes that have previously been shown to co-operate in the development of renal cell carcinoma. This is the first description of a potential direct fusion between these two genes.

## Materials and Methods

### Experimental Confirmation

The validation of ten isoforms from six of the fusion genes identified by FusionFinder was performed by Sanger sequencing of RT-PCR products. Total RNA from K562 ([36] a gift from Prof. GR Shellam, University of Western Australia) was reverse transcribed using gene specific primers (individual details for successful primers can be found in Table S3) and Omniscript RT (Qiagen) according to the manufacturer’s instructions. RT-PCR products were then amplified using GoTaq® Flexi DNA polymerase (Promega). The amplification conditions were as follows: initial denaturation at 95°C for 2 minutes, followed by 35 cycles of 30 seconds at 95°C, 30 seconds at 60°C and 45 seconds at 72°C, with a final extension step of 72°C for 5 minutes. PCR products were gel extracted and purified using a QIAquick Gel Extraction Kit (Qiagen) and were then subject to Sanger Sequencing using BigDye® Terminator V3.1 (Applied Biosystems - ABI). The sequencing amplification conditions were as follows: initial denaturation at 96°C for 1 min, followed by 35 cycles of 10 seconds at 96°C, 5 seconds at 50°C and 4 minutes at 70°C. Samples were purified and then sequenced on an ABI 3130 xl machine. Sequences were aligned to the original fusion transcript sequences for validation.

### Generation of Simulated Datasets

To produce a simulated dataset that was representative of a real SE RNA-Seq run, we firstly examined an in house RNA-Seq dataset to establish what typical proportion of all possible Ensembl genes and transcripts were represented and the distribution of reads representing them. Using these parameters we then wrote a Perl script that randomly selected transcripts from Ensembl and through sampling from our known read distribution, a random number of reads were then generated across the full length of each selected transcript. During this process, pairs of transcripts from different genes were selected at random to be used as fusion transcripts. The sequences of a single exon from each fusion transcript were then combined and a random number of reads were generated across the simulated fusion breakpoint, ensuring that each read contained at least 30 bases of either exon. All normal and fusion reads were generated in the forward orientation with a random number being reversed and complemented to represent the fact either strand of a cDNA fragment may be sequenced. In addition to the normal and fusion reads, 10,000 random sequence reads were generated to represent reads of poor quality sequence.

### Calculation of Performance Measures

For our comparison of software performance, sensitivity was defined as the proportion of correctly identified fusion genes and PPV as the proportion of identified fusion genes that are true simulated fusion genes. These performance measures are defined as follows:

*Sensitivity_i_* 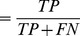


Positive Predictive Value_i_ 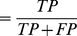


where *TP* (True Positive) is the number of correctly identified fusion genes, *FN* (False Negative) is the number of fusion genes that are not correctly identified and *FP* (False Positive) is the number of genes incorrectly identified as fusion genes. The number of reads evidencing the fusion genes is denoted by *i* and in our simulations ranged from 1 to 295.

## Supporting Information

Table S1 The raw FusionMap (a) and Tophat-Fusion (b) output following an analysis of the Levin dataset.
(XLS)

Table S2 The sensitivity measurements for each of the 55 simulated fusion genes (a) and the raw FusionFinder (b), FusionMap (c) and Tophat-Fusion (d) output.
(XLS)

Table S3 Primers used for gene-specific cDNA synthesis, PCR amplification and sequencing.
(DOC)

Table S4 Performance of each FusionFinder analysis step.
(DOC)

File S1 Conditions for finding intra-exon breakpoints with FusionFinder.
(DOC)

Box S1 A summary of the FusionFinder step-wise analysis protocol.
(DOC)
