# Supplementary material for: FusionFinder: A Software Tool to Identify Expressed Gene Fusion Candidates from RNA-Seq Data
Source: PLoS One. 2012 Jun 27;7(6):e39987. doi: 10.1371/journal.pone.0039987 (PMC3384600; doi:10.1371/journal.pone.0039987)
Supplement: Table S3 — Primers used for gene-specific cDNA synthesis, PCR amplification and sequencing. (DOC) [file pone.0039987.s003.doc]

**Table S3: Successful primers used to experimentally confirm known and novel fusion genes in our K562 cell line**

| **Fusion Gene** | **Isoform** | **PRIMERS** | | |
| --- | --- | --- | --- | --- |
|  |  | **Gene Specific cDNA Synthesis** | **PCR and Sequencing** | |
|  |  |  | **Forward** | **Reverse** |
| BCR:ABL | Isoform1: 23632551-23632600_ 133729451-133729500 | GTTTGGGCTTCACACCAT | CTCCTCTGACTATGAGCGTG | GCCACAAAATCATACAGTGC |
| PRIM1:NACA | Isoform1: 57127931-57127969_ 57108423-57108464 | GTAACTCCTGTAACCTGCCG | TTACTGTTCCGACCATAAGC | CCGAAGACCCAGTTTGGACA |
|  | Isoform2: 57127931-57127972_ 57118262-57118303 | CCTTCCAGTGCCTTCTTT | GCAGAAAGTGGACCAGTTTG | GGCAACTCCTGCTCTGTA |
| C3orf10:VHL | Isoform2: 10167368-10167392_ 10188209-10188238 | CATTTGGGTGGTCTTCCA | CCAGCAGCATCAAGAAAATC | GTAGAGCGACCTGACGATGT |
| ACCS:EXT2 | Isoform3: 44104833-44104861_ 44129250-44129279 | CCCAGGAGGACAATGGAGAA | TGCCCACACCTATGTCTCAG | GCGATAGACATCAAAACACG |
| SLC29A1:HSP90AB1 | Isoform1: 44200122-44200165_ 44216369-44216415 | CAAGGCATCAGAAGCATTAG | AGCCCACCAATGAAAGCCAC | CTTTACCACTGTCCAACTTCG |

The primers used to confirm two of the three novel fusion genes and the additional isoform of the *PRIM1:NACA* fusion gene can be found in rows 3, 4 and 5. Only those primers that gave PCR products are shown. No PCR product was found for the novel fusion gene *CEP170:RAD51L1*, isoform 1 of *C3orf10:VHL* or isoforms 1 and 2 of *ACCS:EXT2*, in our K562 cell line.
