# Supplementary material for: FusionFinder: A Software Tool to Identify Expressed Gene Fusion Candidates from RNA-Seq Data
Source: PLoS One. 2012 Jun 27;7(6):e39987. doi: 10.1371/journal.pone.0039987 (PMC3384600; doi:10.1371/journal.pone.0039987)
Supplement: Table S4 — Performance of each FusionFinder analysis step. (DOC) [file pone.0039987.s004.doc]

**Table S4: Performance of each FusionFinder analysis step**

| **FusionFinder Analysis Step** | **Time taken Single Core** | **Time taken Five Cores** | **Maximum memory usage** |
| --- | --- | --- | --- |
| *Step 1* - ***Alignment of full length reads against a normal coding reference transcriptome using Bowtie*** | 26mins | 6mins | 260MB |
| *Step 2* - ***Creation of pseudo paired-end reads*** | 1hr38mins | 1hr38mins | 1.8GB |
| *Step 3* - ***Alignment of pseudo paired-end reads against a normal coding reference transcriptome using Bowtie*** | 23mins | 5mins | 260MB |
| *Step 4* - ***Analysis and false-positive filtering of the pseudo paired-end read results***  *Step 5* - ***Block filtering and identification of fused exons from candidate fusion transcripts*** | 39mins | 39mins | 255MB |
| Total | 3hrs6mins | 2hrs28mins |  |

Steps 1 and 3 were performed using Bowtie (version 0.12.7). Data based on the analysis of the Levin dataset comprising 14 million 76 mer reads, using either a single core or five cores on a 64-bit linux machine with AMD Opteron 8431 (2.4GHz, four Six Cores) and 32GB RAM.
