# Supplementary material for: FusionFinder: A Software Tool to Identify Expressed Gene Fusion Candidates from RNA-Seq Data
Source: PLoS One. 2012 Jun 27;7(6):e39987. doi: 10.1371/journal.pone.0039987 (PMC3384600; doi:10.1371/journal.pone.0039987)
Supplement: File S1 — Conditions for finding intra-exon breakpoints with FusionFinder. (DOC) [file pone.0039987.s005.doc]

**File S1: Conditions for finding intra-exon breakpoints with FusionFinder**

Fusions involving intra-exon breakpoints (IEB) are not intended to be consistently detected by FusionFinder but will be detected in certain situations.

During the mapping step (4E in the manuscript) FusionFinder allows for the presence of small canonical exons to exist in the expected gap between the two pseudo paired-end reads. Therefore if a truncated IEB exon can masquerade as a short canonical exon it will be regarded as a canonical intervening exon at this step. Only at the subsequent alignment stage will it be discovered as an IEB.

An example of this is outlined below. Here a gene fusion has been detected involving the genes *BRK1* (*C3orf10*) and *VHL*. The IEB is in an exon of the 5' gene partner *BRK1* (*C3orf10*) highlighted in green below. The 5' pseudo paired-end read aligns mostly in the *BRK1* exon upstream of the IEB and spans 5 bases into the IEB exon. The 3' pseudo paired-end read aligns 11 bases downstream of the start of the *VHL* fusion exon. In the example below, for an acceptable mapping to occur the FusionFinder mapping algorithm requires there to be 16 bases between the two 30 base pseudo paired-end reads as they are derived from a parent 76 base read (76-(2*30)=16). Eleven of these bases come from the distance between the start of the *VHL* exon and the start of the 3' pseudo paired-end read. The remaining 5 bases come from the end of the 5' pseudo paired-end read to the start of the *VHL* exon. So this means that for a valid mapping to exist there needs to be a small exon of 10 bases in length (5 bases from the 5' pseudo paired-end read overlapping the IEB exon plus 5 bases between the end of the 5' pseudo paired-end read to the start of the *VHL* exon). In this case the algorithm queries Ensembl for any exon downstream of the *BRK1* exon or any exon upstream of the *VHL* exon that is of the required length (in our case 10bases). It just so happens that as well as the IEB exon being 10 bases, there is an upstream exon of the *VHL* that is 10 bases in length (see Ensembl screenshot below). As a consequence of this coincidence, in this case the mapping was accepted as valid. It is only at the stage of viewing the alignments of these fusions was the IEB detected. Also as a consequence of these lengths being the same, an open reading frame is maintained across the three fused exons.

In summary then for an IEB to be detected both the following conditions would need to be in effect:

1. At least one of the pseudo paired-end reads must map overlapping a canonical exon boundary

2. At least one of the implicated fusion genes (G1 or G2) must have an exon either downstream (G1) or upstream (G2) of the implicated fused G1:G2 exons whose length allows the mapping criteria (ie must be an expected distance between the two pseudo paired-end reads) to be met.

CLUSTAL W(1.81) multiple sequence alignment

G1exon_ENSE00002188501/1-30 GAACGGAGAATAGAGTACATTGAAGCTCGG------------------------------

G2exon_ENSE00001163994/1-30 ----------------------------------------GTCACCTTTGGCTCTTCAGA

BI:081030_SL-XBF_0001_FC30CB2AAXX:7:64:975:161/1-76 -----GAGAATAGAGGACATTGAAGCTCGGGTGACAAAAGGTCACCTTCGGCTCATCAGA

BI:081030_SL-XBF_0001_FC30CB2AAXX:7:56:1209:944/1-76 -----GAGAATAGAGTACATTGAAGCTCGGGTGACAAAAGGTCACCTTTGGCTCTTCAGA

BI:081030_SL-XBF_0001_FC30CB2AAXX:7:20:46:353/1-76 -----GAGAATAGAGTACATTGAAGCTCGGGTGACAAAAGGTCACCTTTGGCTCTTCAGA

BI:081030_SL-XBF_0001_FC30CB2AAXX:7:90:905:390/1-76 -----GAGAATAGAGTACATTGAAGCTCGGGTGACAAAAGGCCACCTTTGGCTCTTCAGA

Expected insert 16bp

G1exon_ENSE00002188501/1-30 ---------------------

G2exon_ENSE00001163994/1-30 GATGCAGGGA-----------

BI:081030_SL-XBF_0001_FC30CB2AAXX:7:64:975:161/1-76 GATGCAGGGACACACGATGGG

5' and 3' pseudo paired-end read sequences

BI:081030_SL-XBF_0001_FC30CB2AAXX:7:56:1209:944/1-76 GATGCAGGGACACACGATGGG

BI:081030_SL-XBF_0001_FC30CB2AAXX:7:20:46:353/1-76 GATGCAGGGACACACGATGGG

BI:081030_SL-XBF_0001_FC30CB2AAXX:7:90:905:390/1-76 GATGCAGGGACACACGATGGG

Figure shows an alignment of all 76bp parent reads providing evidence for a fusion between *BRK1* and *VHL* and the implicated fused exons. Highlighted are the end of one *BRK1* exon (ENS00002188501) in red joined to all 10bp of a truncated *BRK1* downstream exon (ENS00002146984) in green fused to the *VHL* exon (ENSE00001163994) in blue. Also highlighted in yellow are the 5' and 3' pseudo paired-end read sequences derived from the parent 76bp read and the expected insert size.

Figure shows the exonic sequences of the *BRK1* Ensembl transcript ENST00000530758. Highlighted are the implicated *BRK1* (G1) exon ENS00002188501 and the canonical downstream exon ENS00002146984. Also highlighted is the experimentally confirmed intra-exon breakpoint position as predicted by FusionFinder.


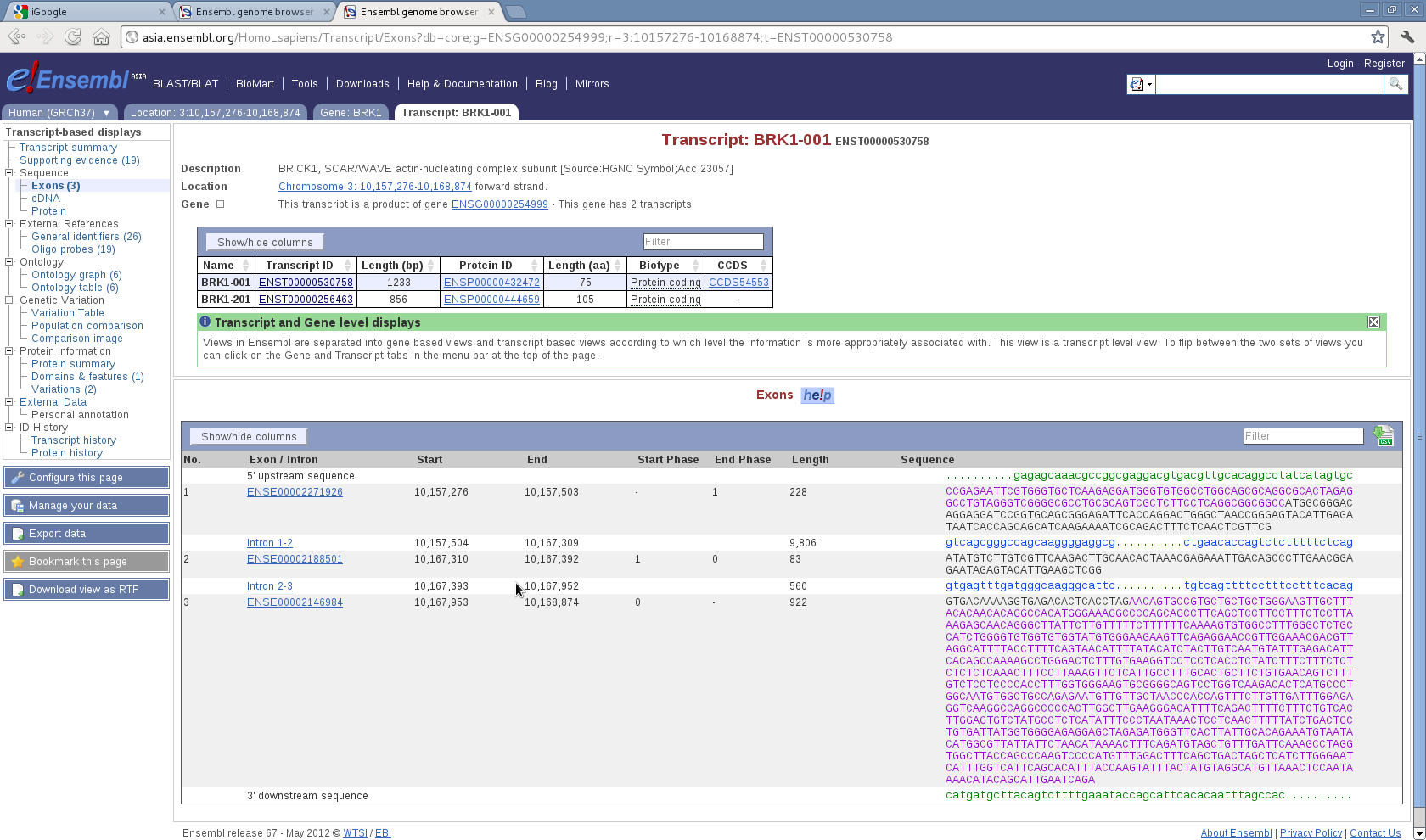


Intra-exon breakpoint position

**Downstream, truncated G1 exon**

**G1 exon**


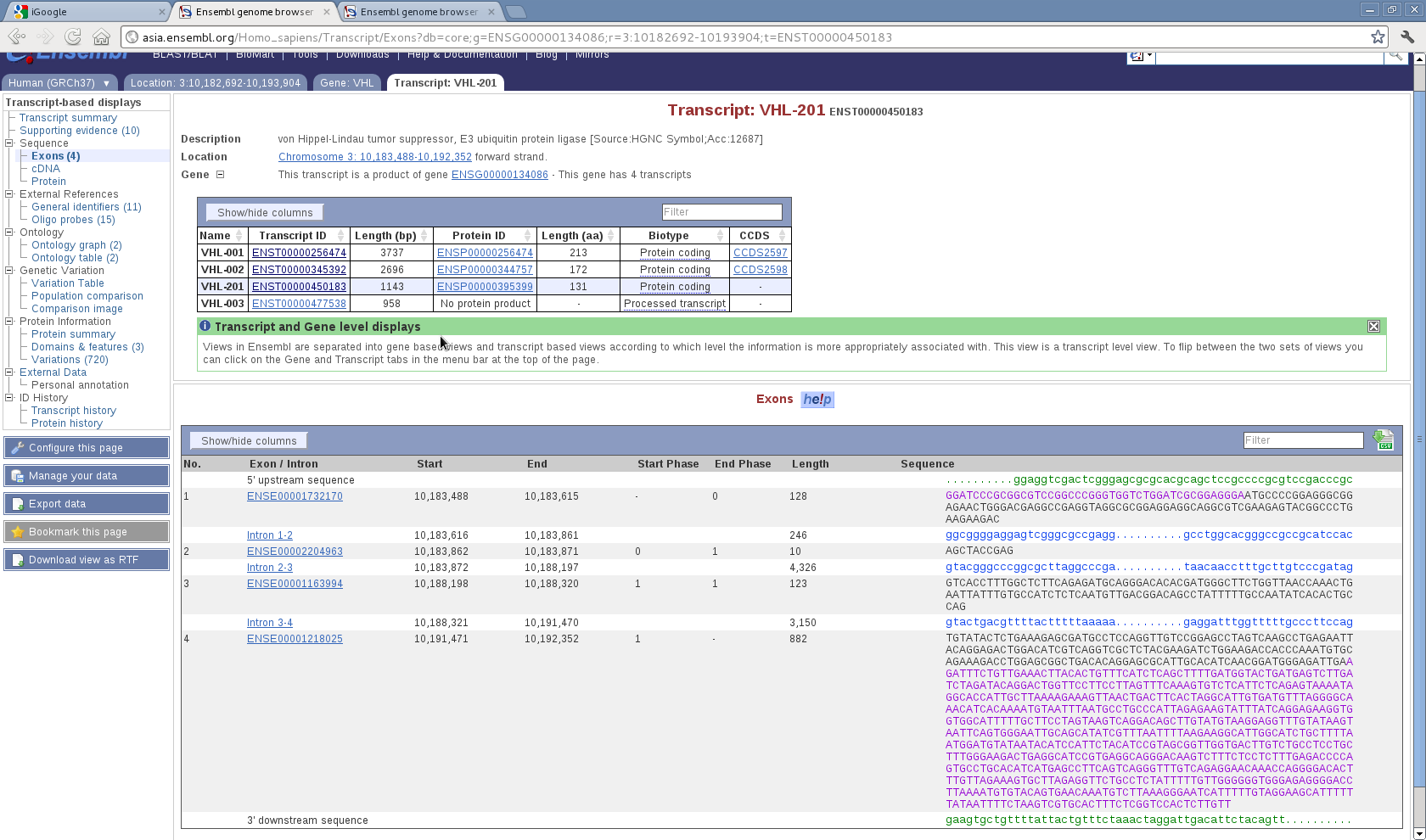


**G2 exon**

Figure shows the exonic sequences of the *VHL* Ensembl transcript ENST00000450183. Highlighted is the implicated *VHL* (G2) exon ENSE00001163994 and the short 10bp upstream exon ENSE00002204963 that facilitated a valid mapping.

Short 10bp G2 exon that permitted a valid mapping
