# Supplementary material for: FusionFinder: A Software Tool to Identify Expressed Gene Fusion Candidates from RNA-Seq Data
Source: PLoS One. 2012 Jun 27;7(6):e39987. doi: 10.1371/journal.pone.0039987 (PMC3384600; doi:10.1371/journal.pone.0039987)
Supplement: Box S1 — A summary of the FusionFinder step-wise analysis protocol. (DOC) [file pone.0039987.s006.doc]

**Box S1: A summary of the FusionFinder step-wise analysis protocol**

*Step 1* - ***Alignment of full length reads against a normal coding reference transcriptome using Bowtie***

Reads successfully mapping to the reference are discarded, non-mapping reads (potentially representing sequenced fusion transcripts) are retained.

*Step 2* - ***Creation of pseudo paired-end reads***

Extraction of smaller sections from either end of each non-mapping read (called pseudo paired-end reads)

*Step 3* - ***Alignment of pseudo paired-end reads against a normal coding reference transcriptome using Bowtie***

Identification of different genes (G1 and G2) mapping to a pair of pseudo paired-end reads (read pair)

*Step 4* - ***Analysis and false-positive filtering of the pseudo paired-end read results***

1. Filter out read pairs where both reads hit the same gene in the reference transcriptome
2. Filter out read pairs where either or both match nothing in the reference transcriptome
3. Optionally filter out read pairs where either or both reads map to paralogous genes
4. Optionally filter out read pairs where the reads hit genes that occur on the same chromosome but on opposite strands (most likely representing antisense transcripts)
5. Obtain the genomic coordinates of the aligned position of the read on the transcript and filter out read pairs that do not map with the expected distance between them
6. Filter out read pairs that represent pseudogenes or non-paralogous genes containing exons found in other genes

*Step 5* - ***Block filtering and identification of fused exons and isoforms from candidate fusion transcripts***

1. For each remaining G1:G2 pair, combine the genomic coordinates of all overlapping reads into "alignment blocks"
2. Filter out alignment blocks where the block on G1 overlaps a repeat of the same class as the block on G2
3. Obtain the Ensembl exon closest to the genomic extremities of each alignment block
4. Optionally filter out G1:G2 pairs that are evidenced by less than a given number of pseudo PE reads
5. Assign categories based on the chromosomes where G1 and G2 lie, their orientation and their proximity
